# Effects of neural stem cell transplantation in Alzheimer’s disease models

**DOI:** 10.1186/s12929-020-0622-x

**Published:** 2020-01-27

**Authors:** Yoshihito Hayashi, Huan-Ting Lin, Cheng-Che Lee, Kuen-Jer Tsai

**Affiliations:** 1 Institute of Clinical Medicine, College of Medicine, National Cheng Kung University, Tainan, Taiwan; 2 Department of Life Sciences, College of Bioscience and Biotechnology, National Cheng Kung University, Tainan, Taiwan; 3 Division of Stem Cell Processing/Stem Cell Bank, Center for Stem Cell Biology and Regenerative Medicine, The Institute of Medical Science, The University of Tokyo, Tokyo, Japan; 4 Research Center of Clinical Medicine, National Cheng Kung University Hospital, College of Medicine, National Cheng Kung University, Tainan, Taiwan; 5 Center of Cell Therapy, National Cheng Kung University Hospital, College of Medicine, National Cheng Kung University, Tainan, Taiwan

**Keywords:** Alzheimer’s disease, Neural stem cell, Synaptogenesis, Neurogenesis, Inflammation, Cognitive impairment, Cell therapy

## Abstract

Currently there are no therapies for treating Alzheimer’s disease (AD) that can effectively halt disease progression. Existing drugs such as acetylcholinesterase inhibitors or NMDA receptor antagonists offers only symptomatic benefit. More recently, transplantation of neural stem cells (NSCs) to treat neurodegenerative diseases, including AD, has been investigated as a new therapeutic approach. Transplanted cells have the potential to replace damaged neural circuitry and secrete neurotrophic factors to counter symptomatic deterioration or to alter lesion protein levels. However, since there are animal models that can recapitulate AD in its entirety, it is challenging to precisely characterize the positive effects of transplanting NSCs. In the present review, we discuss the types of mouse modeling system that are available and the effect in each model after human-derived NSC (hNSC) or murine-derived NSC (mNSC) transplantation. Taken together, results from studies involving NSC transplantation in AD models indicate that this strategy could serve as a new therapeutic approach.

## Introduction

Alzheimer’s disease (AD) is a common progressive neurodegenerative disorder that has been studied by scientists for over a century. It was first named by Alois Alzheimer in 1906 [1]. The symptoms of AD include memory loss and cognitive impairment caused by significant losses in the number of neurons in the cortical and subcortical regions [2]. A large proportion of the elderly population suffers from AD, exacerbating the economic burden associated with an ageing society. Indeed, the number of patients continues to grow and is estimated to double or triple within the next few decades [3]. Therefore, optimizing the treatment for AD is of great priority.

### Models of Alzheimer’s disease

Although the volume of studies that has been undertaken is considerable, elements of the disease mechanism and the relationship of pathological proteins in AD development remain uncertain. Several studies have used AD mouse models to address some of these questions. However, their physiological relevance to humans is questionable, since animal models have yet to fully recapitulate human AD. The dominant hypothesis for AD development is amyloid-beta (Aβ) aggregation in the extracellular region and neurofibrillary tangles caused by tau hyperphosphorylation in the intracellular space. These irregular protein aggregations are followed by neuron degeneration and synaptic loss. Notably, patients with early on-set AD carry only the Aβ mutation, not the tau mutation [4]. In order to closely mimic the intracellular and extracellular microenvironment of patients with AD, it is necessary to introduce additional mutations to genes encoding amyloid precursor protein (APP) and presenilin-1 (PS1), as well as an extra tau mutation into triple-transgenic (3xTg) mice. This extra tau mutation in 3xTg mice has reduced the reliability of the model. Other alternatives include the Tg2576, APP/PS1 and 5xfAD mouse models, but in these instances only Aβ aggregation is observed but no neurofibrillary tangles. Moreover, in mice models, no significant neuron loss or cognitive dysfunction occurs before Aβ deposition as observed in actual AD patients [5, 6]. It remains unclear the extent to which these discrepancies in observation are attributable to the different genetic composition of these mouse models of AD.

More recently, induced pluripotent stem cells (iPSCs) have been derived from patients with AD and established as a disease model. Numerous studies in AD-iPSCs have reported that levels of toxic Aβ and hyperphosphorylated tau protein are dramatically elevated in differentiated neuronal cells. However, no Aβ plaques or neurofibrillary tangles form. This may be due to limitations in the culture system and that differentiated cells have yet to reach mature status. Furthermore, AD-iPSC genotypes vary amongst donors, thus differentiated cells from one individual alone is insufficient to model the abnormal cellular network in AD in its entireity. Additionally, the pathological hallmarks of AD are expressed earlier in AD-iPSCs than in AD patients thus similar to existing mouse models, recapitulation of AD is incomplete. Combined with the wide range of both genomic and phenotypical variations in iPSCs, the suitability of their application as a modelling system remain debatable. As such, fair comparisons can only be made using an isogenic control, which will require complex gene editing techniques to correct the mutations [7].

### Current treatment of AD

**Fig. 1:**
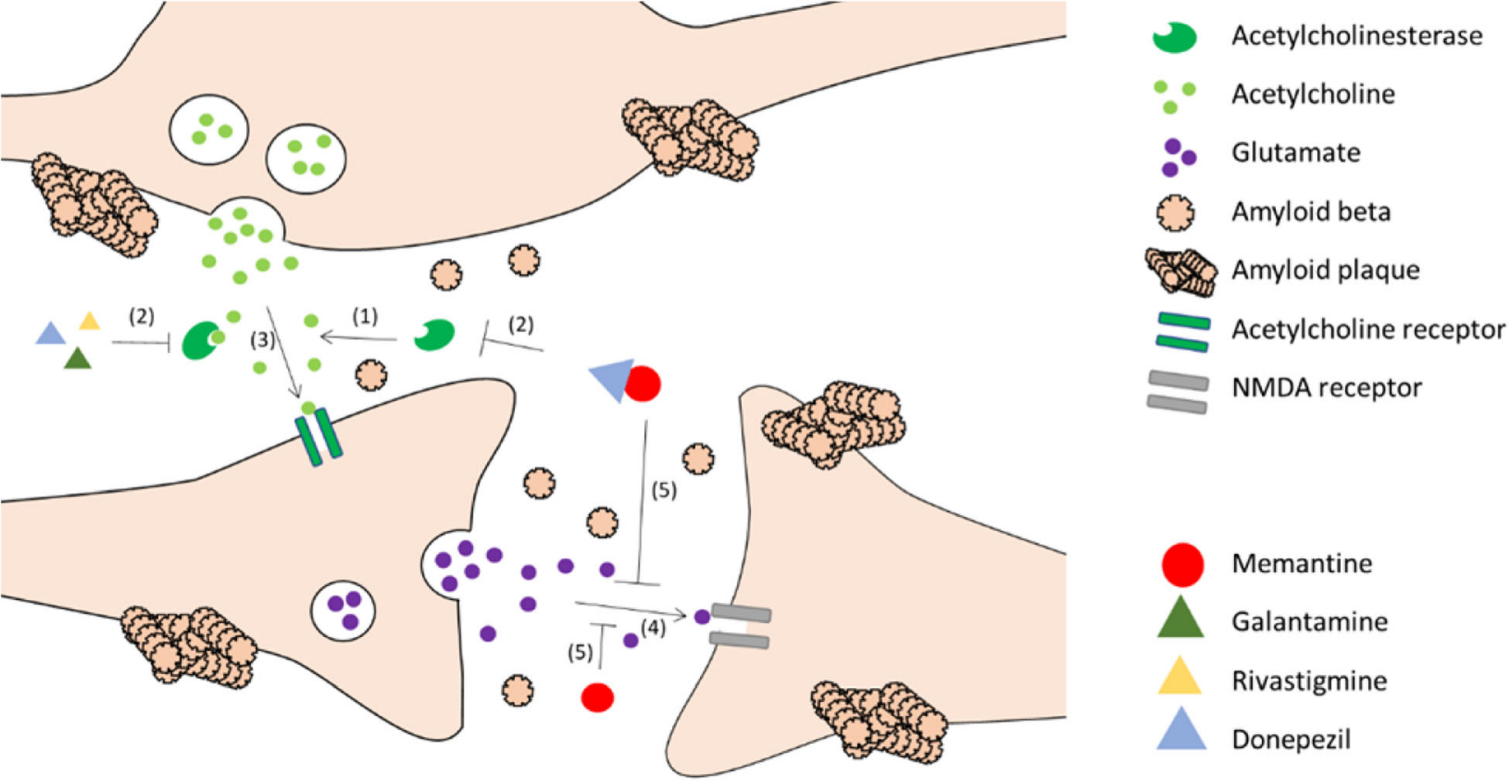
The mechanisms of the respective drugs. Acetylcholinesterase inhibitors (galantamine, rivastigmine and donepezil) enhance the activity of neuro-message transduction by preventing acetylcholine degradation (1,2,3). NMDA receptor antagonists (memantine) compete with glutamate in binding to the NMDA receptor to inhibit Ca^2+^ influx into the postsynapse (4,5). These drugs have little effect on amyloid-beta production and aggregation, synaptogenesis, and neurogenesis yet they rescue cognitive impairment

Reducing Aβ levels has been the dominant treatment strategy in development to halt, retard or even reverse the progression of AD pathology. However, there are no Food and Drug Administration (FDA)-approved drugs targeted at reducing Aβ levels. In fact, no new Alzheimer’s drug therapies have been approved for almost two decades, and only three types of cholinesterase inhibitors, one N-methyl-d-aspartate (NMDA) receptor antagonist, and one combined drug therapy (memantine plus donepezil) are currently approved for clinical use [8]. Donepezil, rivastigmine, and galantamine are cholinesterase inhibitors that reduce acetylcholinesterase activity and thus prevent insufficient acetylcholine levels in the synaptic region. Preserving acetylcholine levels allows effective neuronal function despite pathological protein aggregation. However, excess excitatory stimulation, especially that caused by high glutamate levels, can lead to an excitotoxic microenvironment in the synaptic region through invasive calcium influx. This may eventually damage or even lead to neuronal cell death [9]. Many studies have shown that such hyperstimulation is closely associated with oversensitive NMDA and/or AMPA receptors. The drug memantine, which is an NMDA receptor antagonist, acts to offset this harmful Ca^2+^ influx into neurons [10]. Finally, combination therapy using memantine and donepezil combines the effects of a cholinesterase inhibitor and an NMDA-receptor antagonist (Fig. 1). This combined therapy appears to be more effective [11]. However, it also carries greater possibility of occurrence of more serious side effects such as seizure, slow heartbeats and severe gastrointestinal problems compared with single drug treatment [12]. Thus, it is unclear how valuable such a palliative drug-based approach can be. New drugs that target the pathological protein itself—so-called anti-amyloids medication —are experiencing difficulties in clinical trials [13] as the effects appear independent from symptomatic improvement [14]. Meanwhile, researchers are investigating the potential use of vaccinations to counter plaque formation, as well as more advanced techniques that facilitates early AD diagnosis, which could be especially beneficial to patients before they enter the more severe late stages of the disease [15].

## Therapeutic effect of neural stem cell transplantation

### Neural stem cells

As a novel therapeutic strategy, neural stem cell (NSC) transplantation, which target both neuron networks and pathological proteins, produces beneficial result in behavior and microenvironment. In brief, most traditional drug therapies act merely upon the microenvironment. As multipotent stem cells, NSCs can self-renew and differentiate into various cell types, such as neurons and glial cells [16, 17]. NSCs can be collected from brain tissue, genetically reprogrammed from somatic cells [18, 19], or even differentiated from embryonic stem cells (ESCs) and iPSCs [17, 20]. In adults, NSCs are localized in the sub-ventricular zone (SVZ) and hippocampus [21, 22]. As with drug therapy, many studies have suggested that NSC transplantation improves cognitive behaviour in animal models of AD [23], Parkinson’s disease [24, 25] Huntington’s disease [26, 27], amyotrophic lateral sclerosis [28] and other neurodegenerative diseases. After transplantation, NSCs differentiate into neurons and/or glial cells and release trophic factors. Asymmetric NSC division generates different cell types that replace damaged neurons [29, 30] and the neurotrophic factors released from both differentiated cells and stem cells are related to rapid differentiation [31] and play a significant role in neuroprotection to rescue synaptic density [32–34]. Secretion of neurotrophic factors and cell restoration has been shown to improve individual memory function [35, 36]. Furthermore, modified NSCs overexpress Aβ degrading-enzyme [37], which reduces Aβ aggregation and improves synaptic density. Novel drugs that are currently in development have shifted their focus to targeting these mechanisms to halt or reverse disease progression [38]. Considering that NSCs can restore damaged cells, reduce Aβ aggregation, ameliorate AD pathology as well as restoring neuronal cell populations [32, 34, 39], NSC therapy is a promising and flexible novel therapeutic strategy for targeting the primary cause of AD. Unfortunately the efficacy compared with placebo groups has been inconsistent, not to mention several ethical questions and disagreements on how they should be correctly handled [40]. Nonetheless, stem cell therapy is certainly one of the most promising therapeutic strategies in development.

### Different effects of NSC transplantation in Alzheimer’s models

#### Human-derived NSC vs murine-derived NSC in 3xTg mice

The 3xTg mouse is a triple-transgenic AD animal model established by Oddo et al. in 2003. The model carries three mutations related to familial Alzheim’s disease (FAD): APP Swedish, MAPT P301L, and PSEN1 M146 V. The 3xTg mouse model is the first transgenic AD model to express both Aβ aggregation and neurofibrillary tangles from hyperphosphorylated tau protein. Intracellular and extracellular Aβ aggregation is observed at 4 months and 6 months of age respectively, while cognitive impairment starts at 4 months and tau is first observed at 12 months [41, 42].

In 2015, Ager et al. first introduced human central nervous system stem cells (hCNS-SCs) into 3xTg mice. The transplanted hCNS-SCs differentiated into NSCs and then into immature neurons and glial cells, which improved synaptic density. Although the levels of Aβ and tau proteins remained unchanged, both the Morris-water-maze and novel object recognition tests indicated improved memory consolidation. In contrast, no significant improvement in learning ability was observed after hCNS-SCs transplantation. Although encouraging, these results suggest that specific differentiation into neuronal cell lineages alone contributes little to cognitive recovery, and that hCNS-SC transplantation may serve to reverse the symptoms only [43] (Table 1).

**Table 1:** Summary of factors and effects after neural stem cell transplantation in 3xTg mice

NSC	Region	Factor	Effect	Not-shown	Aβ/tau	Ref
3xTg mice						
hCNS-SC	Hippocampus	↑immature neuron ↑immature glia cell ↑synaptic density	↑endogenous synaptogenesis	・The relation of endogenous synaptogenesis and hCNS-SC ・Role of neurotrophic factor	×	[43]
GFP tg mice	relative to Bregma of: AP:_ 2.06, ML:_1.75, DV:_1.75	↑BDNF	↑synaptic density	・Axonal growth in vivo	×	[23]
GFP-C57BL/6 mice	hippocampal CA1	NSC	↑neuronal regeneration	・Origin of newly synthesized neuron ・mechanism of neural regeneration	△	[44]
GFP tg mice	hippocampus subiculum	NSCs delivered NEP	↑synaptic density ↓Aβ	・Link between Aβ level and cognitive deficit	〇	[45]

**Key:** 〇 changes, △ not mentioned, × unchanged

Interestingly, transplanting mNSCs instead of hNSCs produced similar results in the 3xTg mice model. In a study by Mathew et al., both neurotrophin and brain-derived neurotrophic factor (BDNF) secreted from transplanted NSCs enhanced synaptic density and rescued cognitive impairment. However, this result was again independent from Aβ and tau levels. In the same study, BDNF was shown to support axon growth in vitro thus increasing synaptic density [23]. Furthermore, cell regeneration and/or repair triggered by NSCs improves cognitive function by ameliorating neuronal networks [44], so NSCs are closely associated with improved behavioural performance in the 3xTg animal model. To further evaluate the impact of NSCs under conditions of pathological protein alteration, modified NSCs carrying Neprilysin (NEP) were introduced into 3xTg mice. Viral vector-delivered NEP was then compared with NSC-delivered NEP and found to be less widely distributed throughout the brain. Moreover, peripheral NEP introduction had less effect in clearing Aβ in the brain. These results suggest that NSCs can act as an effective NEP-delivery vehicle. It follows that the combination of NEP delivery and NSC transplantation further improves synaptic density by decreasing Aβ levels, and that NSCs may be a promising AD therapeutic strategy [45, 46] (Table 1).

Neurotrophin release and neurogenesis in 3xTg mice is highly dependent on the source of NSCs. Specifically, in Ager’s study, hNSCs differentiated into immature neurons and glial cells and induced endogenous synaptogenesis. Growth-associated protein 43 (GAP-43) is located in the axon to support synapsis and neurite stretching. Interestingly, Ager found that following transplantation, GAP-43 was not elevated in the 3xTg model [43], thus it is not yet clear how trophic factors from hNSCs affect synaptogenesis in the 3xTg model. In contrast BDNF, a member of the neurotrophin family of growth factors, from mNSCs could be involved in the recovery of synaptic connectivity [23, 45]. The specificity in NSC differentiation to mature cells and hence the subsequent effect of that has been contradicting. Studies involving hNSCs suggest that lineage-specific differentiation has little effect on cognitive improvement [43], whereas those involving mNSCs suggest that cognitive improvement depends on the precise differentiation of the stem cells to allow cell replacement [44]. Moreover, the potential role of stem cells as vehicles for secreting degrading enzymes has yet to be thoroughly examined in hNSCs. Although improved behavioural performance and cellular changes are observed following transplantation of both hNSCs and mNSCs, the secretory effect and role of hNSCs remains poorly understood (Table 1).

#### Human-derived vs. mouse-derived NSCs in Tg2576

Unlike the 3xTg model, Tg2576 mice only overexpress human Swedish APP (isoform 695; KM670/671NL). These mutations lead to a dramatic increase in Aβ production at about 6 months of age and consequent plaque formation at 9–12 months. Behavioural impairment is observed at 9 months, but some studies have suggested that the mice have no significant behavioural deficit [47]. Mice show no neurofibrillary tangles or significant neuronal loss, but they display progressive pathological protein accumulation and behavioural impairment in many studies, thus partially satisfying the requirements of a typical AD mouse model [6, 48, 49].

Lilja et al. transplanted hNSCs into Tg2576 mice treated with phenserine, which inhibits acetylcholinesterase and Aβ production by lowering expression of APP, an α7 nicotinic receptor (nAChR) agonist, and JN403. In doing so, they could investigate the combined effect of NSCs and drug therapy and found that NSC transplantation was sufficient to trigger endogenous neurogenesis. In the transplant region, many α7 nAChR-expressing astrocytes were found, suggesting that such astrocytes are involved in repairing damaged neurons and growth of new ones. Despite combined treatment using both drugs and NSCs, positive effects such as neurogenesis and cognition recovery was not sustained [50] (Table 2).

**Table 2:** Summary of factors and effects after neural stem cell transplantation in Tg2576

NSC	Region	Factor	Effect	Not-shown	Aβ/tau	Ref
Tg 2576 mice						
hNSCs	Hippocampal DG	↑α7 nAChR-expressing astrocytes	↑Endogenous neurogenesis	・level of Neurotrophic factor ・Synaptic density	×	[50]
Feral cerebral cortex of pregnant C57BL/6 mice	Hippocampal DG	↓β-secretase ↑Neprilysin	↓Aβ production ↓phosphorylated-tau ↑Aβ clearance ↓ pro-inflammatory cytokine ↓inflammatory microglial activation ↑anti-inflammatory cytokines, ↑endogenous neurogenesis ↑synapse formation	・The link between microglia and NSC ・level of BDNF	〇	[51]

**Key:** 〇 changes, △ not mentioned, × unchanged

In the same animal model, following mNSC transplantation at an early stage (13-month-old), changes in both pro- and anti-inflammatory cytokine levels significantly influenced Aβ production and clearance rate by altering enzyme expression in microglial cells. Furthermore, NSCs triggered increases in VEGF, endogenous neurogenesis, and synaptic density, leading to improvements in behavioural performance. However, the same result was not obtained after late-stage (15-month-old) transplantation [51] (Table 2), suggesting that timely intervention is important.

As described above, both hNSCs and mNSCs can initiate endogenous neurogenesis. Notably mNSCs alter microglia from a pro-inflammatory state to an anti-inflammatory state, leading to a decrease in Aβ level through an increase in NEP and phosphorylated tau levels. These effects have yet to be shown in hNSC studies [50, 51] (Table 2).

#### Human-derived vs. mouse-derived NSCs in APP/PS1 mice

APP/PS1 mice are one of the most commonly used AD mouse models. The human APP gene with both Swedish mutation and PSEN1 (L166P) mutation is incorporated into this model. This inserted human gene produces more Aβ than murine APP. Both Aβ 42 and Aβ 40 levels increase with age, yet the ratio of AB42/40 decreases after plaque formation. Aβ aggregates in the neocortex at the age of 6 weeks and in the hippocampus at about 3–4 months [5, 52].

Li et al. transplanted hNSCs into this model and found that the treatment promoted synaptic formation without altering Aβ levels. Some introduced hNSCs differentiated into neural cells in the central nervous system. Indeed, hNSC transplantation enhances neural metabolic activity by increasing both N-acetylaspartate, as seen after medical treatment, and glutamate, a major neurotransmitter related to cell viability and synaptic plasticity [53]. In 2018, a study by McGinley suggested that transplanted hNSCs regulate microglial activation and thus reduces Aβ levels. Furthermore, the beneficial effect of the treatment on behaviour lasted for 4 months after transplantation [54] (Table 3).

**Table 3:** Summary of factors and effects after neural stem cell transplantation in APP/PS1 Tg mice

NSC	Region	Factor	Effect	Not-shown	Aβ/tau	Ref
APP/PS1 tg mice						
hNSCs	Hippocampus	hNSCs N-acetylaspartate, Glu	↑synaptic density ↑Neuronal metabolism	・Level of neurotrophic factor ・inflammatory cytokine	×	[55]
hNSCs	Fimbria fornix	hNSC microglial activation	↑microglia activation ↓Aβ No change in synaptic density No change in Choline acetytransferase	・Roles of neurotrophic factor in long-term effects. ・Anti-inflammatory cytokine ・Factors affects NSC lifespan	〇(AB42 only)	[54]
Non-Tg B6C3 mouse embryos	Hippocampus	Synapses increase	NSC proliferation ↑ synaptophysin and growth factor	・Factor contributes to synaptogenesis. ・synapse function.	△	[56]
Non-Tg B6C3 mouse embryos	Hippocampus	NSC	↑mitochondria ↑mitochondrial-related protein ↓mitochondrial fusion factor optic mitofusion 1&2.	・Synapsis density ・Level of neurotrophic factor	△	[57]
Non-Tg B6C3 mouse embryos	Hippocampus	NSC	↓GFAP, Iba-1, TLR4 and TLR4 etc. ↓proinflammatory mediators	・Synaptic density ・Neurotrophic level	×	[58]
GFP Tg C57BL/6 mice fetal forebrain	Hippocampus	CNL from NSC	↑ChAT mRNA, ↑ChAT activity ↑ACh concentration	・Inflammatory factors	×	[59]
non-Tg B6C3 mouse	Hippocampus	NSC	↑long-term potentiation ↑neuron expressing protein ↑synaptogenesis ↑ BNDF	・Inflammatory factors	×	[60]

**Key:** 〇 changes, △ not mentioned, × unchanged

In another study, mNSCs transplanted into APP/PS1 mice led to a variety of effects, including an increase in synaptophysin and GAP-43, which were in turn associated with an improvement in behaviour accompanied by synaptic formation [56]. In another study, mNSC administration induced BDNF and tropomyosin receptor kinase B (TrkB) release. Furthermore, introduced mNSCs differentiated into neurons to compensate for damaged endogenous neurons. In mNSC-derived neurons, TrkB was highly expressed and enhanced the effect of BDNF upon damaged regions. A protein related to memory and learning function—the NMDA receptor 2B subunit—is also highly expressed after transplantation, leading to cognitive improvement [60]. In addition mNSC-derived cholinergic-like neurons, crucial players in neurotransmition, were also transplanted into the same Tg mice model. As a result, cholinergic acetyl transferase (ChAT) mRNA and protein were upregulated, with an increase in ChAT activity and concentration as well as increased functional synapse density. This result has further inspired efforts to develop NSC treatments since it addresses both molecular and cellular aspects of AD [59]. Zhang et al. investigated alterations in inflammatory activity after mNSC transplantation and found that the activity of glial cells and astrocytes was decreased after mNSC transplantation. This affected the Toll-like receptor 4 signalling pathway and reduced the neuroinflammatory response via a cascade reaction. Cognitive improvement was also observed in the study [58]. Although few of these studies tackled the issue of Aβ levels, they still achieved improvements in behavioural performance via synapsis attenuation (Table 3).

Some partially contradicting results have been obtained. In one study, hNSCs rescued cognitive deficits without altering synaptic density [54], while in another, hNSCs improved synaptic density and neural metabolic activity, but mitigated behavioural improvement [53]. In some studies, hNSC transplantation activated microglia and decreased Aβ level [54], while a review of mNSC studies found no change in Aβ levels, although cognitive deficits were rescued. The decrease in pro-inflammatory factors [58], neuron replacement, increase in cognitive related protein [60] and rise in effective neuronal transmitter levels [59] contributed to this outcome. In contrast, no studies into hNSCs have yet directly investigated the role of neurotrophic factors, so mNSCs have been more thoroughly investigated than hNSCs, even though both hNSCs and mNSCs yield similar results at the behavioural level. Although the precise mechanisms remain controversial, some form of beneficial effect remains consistent throughout (Table 3).

#### Human-derived NSCs in immune-deficient mice

5xfAD mice carries a total of five mutation namely, human APP- the Swedish (K670 N/M671 L), Florida (I716V), London (V717I), PSEN1, M146 L and L286 V mutations. Amyloid-beta aggregation begins to occur at 6 weeks of age and extracellular amyloid deposition starts at 8-weeks age [61]. Spatial and memory impairment is observed in 3 to 6-month ages and continues to worsen [62]. These mice lack the primary constituent cells of adaptive immunity namely T cells, B cells and natural killer cells. This facilitates longer persistence of transplanted NSCs, which will allow long-term efficacy and safety to be evaluated.

When a clinical grade hCNS-SC line was transplanted into 5xfAD mice, successful engraftment had been observed up to five months after transplantation. However, these transplanted hNSC failed to differentiate into neuronal cells and had impact on synaptic density. Pathological protein levels Aβ and BDNF remain unchanged and behavior impairment was not mitigated [63]. In 2019, Zhang et al. transplanted iNPCs reprogrammed from human mononuclear cells into RAG-5xfAD. In this instance, rapid differentiation into neurons and astrocytes were observed. Furthermore, these differentiated neurons formed functional interaction with the host neuron, which rebuilt synapses. An increase in BDNF levels was also observed in the hippocampus. Furthermore, behavior improvement was observed at around 5 to 6 moth post-transplantation [64]. It is worth noting that the source of NSCs from these two studies are very different, where it is plausible to think that reprogrammed somatic cells will have greater neural differentiation capacity. This appears to be the biggest difference between the two studies, thus suggesting that lineage specific differentiation into the desired cell type will have significant effects upon the desired outcome (Table 4). It is known that the adaptive immune system and T cells in particular have a significant role in propagating the neuroinflammatory response [65]. As such, although long-term engraftment of transplanted NSCs was observed, like other mouse models, the accuracy of 5xfAD in modeling AD is also questionable.

**Table 4:** Summary of factors and effects after neural stem cell transplantation in 5xfAD (long-term/about 5 month)

NSC	Region	Factor	Effect	Not-shown	Aβ/tau	Ref
5xfAD mice (Long-term/about 5 month)						
hCNS-SCs	Hippocampus	hCNS-SCs	No change in synaptic density No differentiated neuron No change BDNF No behavior improvement	・Inflammatory factors ・Neurogenesis	×	[63]
iNPCs	Hippocampal DG	hCNS-SCs	↑synaptic density ↑ Neuron ↑ BNDF Behavior improvement	・Inflammatory factors ・Effect of respective cell type after neural cell restore. ・Neurogenesis	〇	[64]

Key: 〇 changes, △ not mentioned, × unchanged

### Mechanisms of behavioral improvement with different NSC sources

#### Role of hNSCs in Tg models

Across the 3xTg, Tg2576, and APP/PS1 Tg mouse models, similar behavioural and cellular effects are produced after hNSC transplantation. In 2015, Ager et al. transplanted hCNS-SCs into the 3xTg model and found that the cells differentiate into immature oligodendrocytes, immature neurons, and a few astrocytes. Their study suggested that NSCs from hCNS-SC trigger endogenous synaptogenesis, leading to cognitive improvement. Additionally, they proposed that specific differentiation stage has little relevance in the improvements seen. Instead, they claimed that the intrinsic properties of hCNS-SCs play an irreplaceable role [43]. Similar behavioural improvement is achieved after endogenous neurogenesis, which is enhanced after hNSC transplantation in Tg2576 mice [50]. Likewise, in APP/PS1 Tg mice, synaptic density and cognitive impairment were significantly improved, and neural metabolism was also ameliorated, suggesting that NSCs may alter neuronal metabolic activity [53]. This was not mentioned in the 3xTg and Tg2576 models. Conversely, another study showed that NSC transplantation has no effect on synaptic density, but that it does improve behaviour [54]. An opposite long-term result in transplanting hNSC into RAG-5xfAD were obtain from two studies. Nonetheless both studies show NSCs have successfully engrafted into the host for at least 5 months. Zhang’s study suggests NSCs differentiation triggers beneficial effect including increase in synaptic density, neural cell number, behavior improvement [64] whereas Marsh’s fail to terminally differentiate NSCs [63]. This information has complicates the causal link and mechanism between NSCs and behavioural improvement, which are nonetheless closely correlated. Interestingly, many studies across different models have implied that NSC transplantation does not alter Aβ levels, while only the study on the APP/PS1 model mentioned microglia-mediated neuroinflammation (Table 5).

**Table 5:** Mechanisms of behavioral improvement after neural stem cell transplantation

	hNSC			mNSC		
	Synaptogenesis	Neurogenesis	Inflammation	Synaptogenesis	Neurogenesis	Inflammation
3xTg	**○**	△	△	**○**	**○**	△
Tg2576	△	**○**	△	**○**	**○**	**○**
APP/PS1	**○**	△	**○**	**○**	△	**○**
RAG-5xfAD^*^	**○/×**	**○/×**	△	△	△	△

^*^Long-term study Key: 〇, changes; △, not mentioned; ×, unchanged

#### Role of mNSCs in Tg models

Generally, mNSC transplantation rescues synaptic density, leading to behavioural improvement in learning and cognition. Together with the 3xTg and Tg2576 mice, mNSC transplantation in APP/PS1 mice triggers synaptic formation. However, in APP/PS1 mice, neurogenesis has not been investigated. In both Tg2576 and APP/PS1 mice, inflammatory level is altered after the transplantation. Notably, NEP produced from microglia in Tg2576 decreases Aβ levels [51], while NSC transplantation in APP/PS1 lowers microglial levels [58]. Neurotrophic factors such as BDNF are elevated in 3xTg mice and APP/PS1 Tg mice, but not in Tg2576 mice. Based on these studies, either neurogenesis or synaptic density is enough to rescue part of the cognitive deficit. Aβ appears to play little role in behaviour, and both Tg2576 and APP/PS1 mice appear sensitive to NSC-mediated neuroinflammatory changes (Table 5).

### AD iPSCs model

Cells with self-renewal and multi-potency characteristics are ideal platforms for drug screening. For instance, iPSCs are associated with fewer ethical concerns and AD-iPSCs models have successfully recapitualted pathological condition for use in novel drug screening such as the combination of bromocriptine, cromolyn, and topiramate as an anti-Aβ cocktail [66], 훾-secretase and β-secretase inhibitors [67, 68]. These drugs inhibit Aβ production and so toxic Aβ level decreases. Especially in anti-Aβ cocktail treatment, toxic Aβ level decrease by more than 60% and which has same result as in inhibitors treatment [66]. Moreover, although FAD and sporadic AD neuron carry different mutations, decrease in Aβ levels was observed in both [67, 68]. Treatment of anti-Aβ antibodies to iPSC-derived neurons have indicated Aβ as upstream of tau hyperphosphorylation. This result further supports known mechanisms and provides clues in drug development [68]. Although behavioural tests cannot be carried out in cell models, iPSCs model could offer significant contribution in elucidation of pathophysiological mechanisms as well as drug screening.

## Challenges surrounding NSC transplantation

Although the potential of NSC therapy is promising, the process of developing it as a treatment for AD is similar to any other drugs. To begin with, it is necessary to clearly establish the positive impact that it could have on patients. However, considering the shortcomings of various AD models, it remains unclear how given outcomes will translate into human patients. Furthermore, although beneficial, the contrasting effect of NSC in different transplantation settings further obscures the definite role of NSCs in therapy. Thus, a comprehensive evaluation of NSC transplantation into AD models will be required.

Many studies have associated cognitive improvement with increases in synaptic density, which is closely related to increases in both neuron and glial cell number. NSC transplantation supports both behavioral and cognitive improvement. However, the exact attributing cell type that supports these improvements that NSCs will need to differentiate into remains unclear. Although NSC transplantation rescues synaptic damage and is involved in functional interaction with the endogenous neuronal circuit, few studies have addressed the duration of this effect. It remains to be seen to what extent an improvement in synaptic density is only a “one-hit” effect or something that is more prolonged in the fight against disease progression towards a cure for AD.

Aβ levels are closely related to the activity of glial cells, which are in turn related to the extent of the inflammatory response. In many studies, various neurotrophins and cytokines act as inducers to promote cell protection or the production of Aβ-degrading enzymes. In fact, NSCs could be genetically modified to highly express Aβ-degrading enzyme and to proliferate widely. Ideally, continuous production of neurotrophic and degrading enzymes by NSCs would prevent further neurodegeneration as disease progresses. However, in practice, Aβ clearance may have little effect on global improvement, because the basal environment remains favorable for Aβ production and aggregation. Thus, to augment the effects of NSC, the basal environment should first be manipulated by influencing local glial cell activity, followed by evaluating transplanted NSCs in terms of clearance and production rates, inflammation level, and neurogenesis.

Localization within the transplant area and viability of transplanted cells are the preliminary challenges in NSC treatment. Subsequent interactions with cells in the host environment is also important. In some studies, NSCs are untraceable after transplantation, while studies with traceable NSCs have not quantified the viable cell number. Methodological difficulties have limited the understanding of NSCs in vivo [69, 70]. The problem of untraceable NSCs in studies post-transplantation has yet to be fully studied. Indeed, there is the inherent risk of transplanted NSCs developing into brain tumor such as glioblastomas. Many studies have identified that cancer stem cells share many common features and niches with NSCs and implicate NSCs as the origin these cells [71]. However, the exact mechanism of how NSCs develops into cancerous cells remains to be elucidated [72].

Ethical concerns around the sourcing of embryonic stem cells, which can be differentiated into NSCs, have continued to persist. However, direct isolation of NSCs from primary tissue is extremely risky, and non-patient-specific NSCs may cause immune rejection. Using iPSCs as an alternative avoids many of the ethical concerns associated with embryonic stem cells. Nonetheless, to what extend these iPSCs are uniformed in their quality remain in question. Moreover, during iPSCs establishment, reprogramming efficiency is depended on donor-cell-type and reprogramming method [73, 74]. The optimum somatic cell type for reprogramming into iPSCs and subsequent differentiation into NSCs remains to be determined. Nonetheless, iPSC-derived NSCs represents a more readily available source of transplantable cells that can be further modified to enhance the beneficial effect of transplantation.

**Fig. 2:**
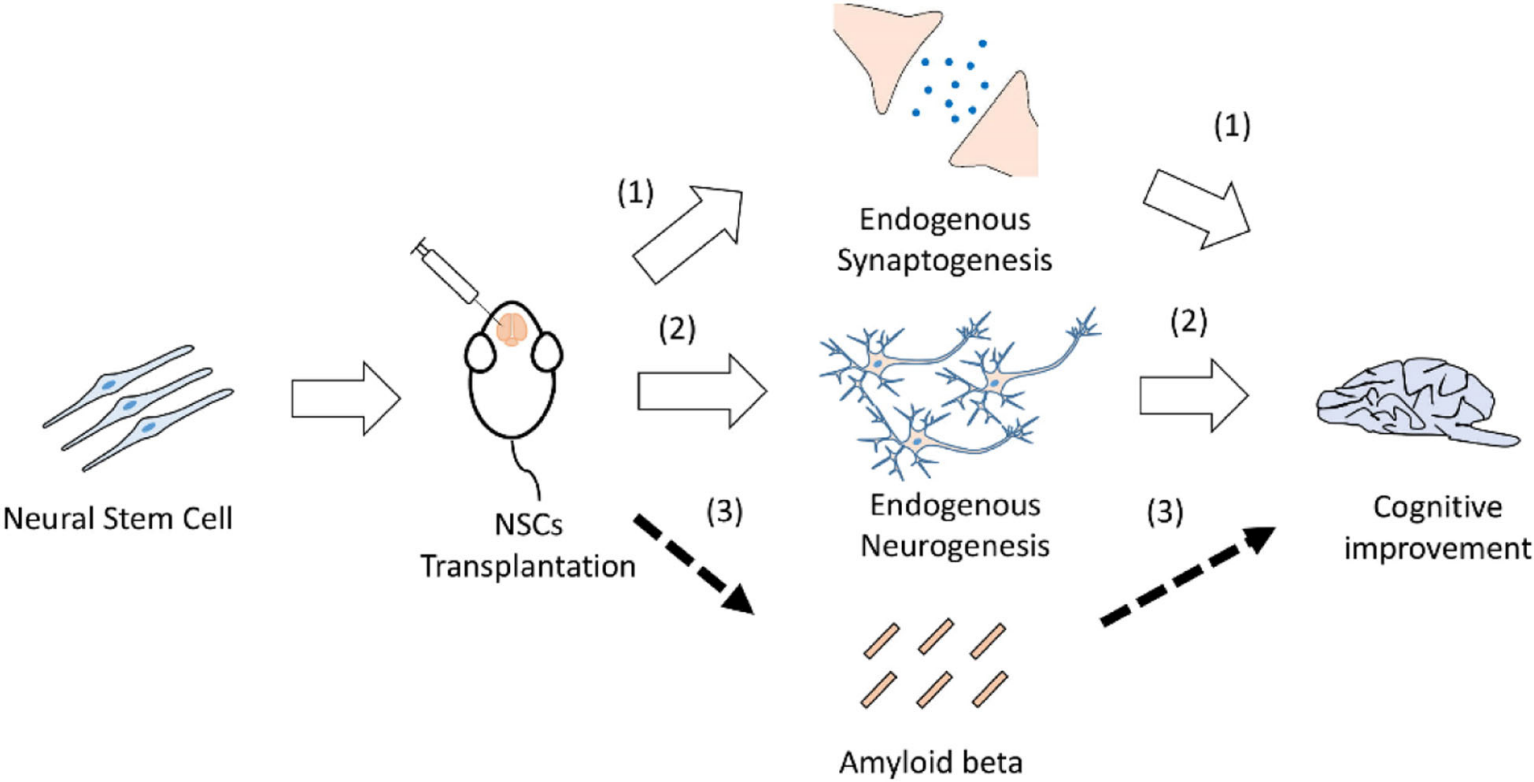
Routes for neural stem cell transplantation and mechanisms of cognitive impairment restoration. Transplantation of neural stem cells triggers (1) endogenous synaptogenesis and (2) endogenous neurogenesis to influence behavioral performance. (3) The limited causal relationship between amyloid-beta and neural stem cells contraindicates any link between behavioral performance and amyloid-beta aggregation

To conclude, the beneficial effect of NSCs is based less on modulating pathological protein levels but rather, increasing synaptic density, restoring local neuron populations, and/or increasing neurotrophic factor levels (Fig. 2). The question is how long can this phenomenon persist for whilst levels of pathological protein level remain unchanged. Also, it would be interesting to know what role NSC can play in lesion protein aggregation through mediating glial cell, inflammation, and synapse rescue. All in all, although certain challenges remain, NSCs will likely have an important role in advancing treatment for AD.

### Recent clinical developments in treatment of Alzheimer’s disease

Aβ related toxicity is still believed to be the main cause of synaptic dysfunction and the subsequent neurodegeneration that underlies the occurrence and development of AD.

Aducanumab is a monoclonal antibody targeting aggregation of Aβ. When transplanted intoTg2576 mice, dose-dependent reduction in both soluble and insoluble Aβ could be occurred and similar observations in a phase 1b randomized trial [75]. To follow up, two identical phase III trials (ENGAGE and EMERGE) were initiated but unfortunately both were discontinued in March 2019 after failing futility testing. Data was re-analyzed to include those who had completed the 18-month follow-up between the futility analysis and halting both studies [76]. In a surprise announcement in October 2019, a new filing for approval for Aducanumab will be made to the FDA. However, experts in the field are being prudent over interpretation of the results as only one of the trials showed moderate benefit in cognitive improvement whereas the other trial still showed no hint of efficacy [77]. Similar observation was observed in phase III trials for Solanezumab, which is also targets Aβ aggregation [78].

Recently in China, conditional approval has been granted for Oligomannate, which aims to prevent neuroinflammation that can occur through stimulated differentiation and proliferation of T helper 1 (Th1) cell by gut dysbiosis [79]. When administered to patients with mild to moderate AD in a phase III study, significant cognitive improvement could be observed compared to the placebo group. However, at the time of writing this review, data for the study has yet to be published. For now, gut dysbiosis and neuroinflammation remains unproven as an effective in in combating AD progression. Many unanswered questions remain for those suffering from more severe forms of AD beyond moderate levels. Cognitive improvement remains the gold standard by which the efficacy of various targeted therapies is judged by. Yet it appears targeting only a single element of AD pathophysiology, such as Aβ accumulation or neuroinflammation will not be enough to arrest disease progression.

## Conclusion and future aspects

Various animal models have been established and each has its own advantages. None have successfully replicated the complex microenvironment of the human brain or the precise pathophysiological conditions of AD. Consequently, it is challenging to precisely characterize the beneficial effects of NSCs in AD. However, it has been consistently shown that transplantation of NSCs does bring some positive effects albeit the mechanisms remains unclear. The number of variable factors remains high in each mouse model, but they fail to compensate for one another. By comparing hNSCs and mNSCs, only a few studies have suggested that Aβ levels in these animal models decrease after hNSC transplantation. Thus, knowing the primary cause of AD is highly due to Aβ aggregation, the functional and characteristic differences in the two types of NSC must be re-examined. Additionally, temporary recovery of behaviour is relatively easily obtained, but often fail to linked to a complete cure. Curative treatment is likely dependent upon sufficiently early diagnosis to prevent further cell death and brain deterioration. A combination of NSC transplantation alongside administrating existing approved medication and preventing further Aβ aggregation may way be the most effective. It important to note that whilst behavioral or cognitive improvement is interpreted as positive outcomes, it can be frequently misinterpreted as permanent arrest or even reversal of AD progression. It merely provides some clues to future treatment thus the focus should shift towards how to sustain such phenomena and combine different treatment that may give rise to such outcomes.

## Data Availability

Not applicable.

## Abbreviations

3xTg: Triple-transgenic
AD: Alzheimer’s disease
APP: Amyloid precursor protein
Aβ: Amyloid-beta
BDNF: Brain-derived neurotrophic factor
ChAT: Cholinergic acetyl transferase
ESCs: Embryonic stem cells
FAD: Familial Alzheim’s disease
FDA: Food and Drug Administration
GAP-43: Growth-associated protein 43
hCNS-SCs: Human central nervous system stem cells
hNSCs: Human-derived neural stem cells
iNPCs: Induced neural progenitor cells
iPSCs: Induced pluripotent stem cells
mNSCs: Murine-derived neural stem cells
nAChR: Nicotinic acetylcholine receptor
NEP: Neprilysin
NMDA: N-methyl-d-aspartate
NSCs: Neural stem cells
PS1: Presenilin-1
SVZ: Subventricular zone
TrkB: Tropomyosin receptor kinase B
